# Interventions with mitochondrial-related effects in older adults with type 2 diabetes: Protocol for a systematic review with conditional network meta-analysis

**DOI:** 10.1371/journal.pone.0360683

**Published:** 2026-10-16

**Authors:** Hao Liu, Aochuan Sun, Yu Deng, Yihan Li, Jiayu Hu, Zhengtang Liu

**Affiliations:** 1 Xiyuan Hospital, China Academy of Chinese Medical Sciences, Beijing, China; 2 Faculty of Chinese Medicine, Macau University of Science and Technology, Macau, China; UNAM Facultad de Estudios Superiores Zaragoza: Universidad Nacional Autonoma de Mexico Facultad de Estudios Superiores Zaragoza, MEXICO

## Abstract

**Introduction:**

Mitochondrial dysfunction is central to type 2 diabetes mellitus (T2DM) pathogenesis, driving insulin resistance and pancreatic β-cell dysfunction. Age-related mitochondrial impairment worsens this in older adults, yet evidence on mitochondrial interventions for this group is limited. This protocol outlines a systematic review and conditional network meta-analysis of randomized controlled trials evaluating mitochondrial interventions in adults aged 60+ with T2DM.

**Objective:**

The primary objective of this systematic review and conditional network meta-analysis (NMA) is to evaluate the comparative efficacy and safety of diverse mitochondrial-related interventions, including pharmacological agents, nutritional supplements, and lifestyle modifications, relative to placebo, standard care, or no intervention, on mitochondrial function biomarkers in adults aged 60 years or older with type 2 diabetes mellitus (T2DM). Interventions with mitochondrial effects are operationally defined as those whose primary mechanism is known to target mitochondrial pathways or those that are explicitly evaluated for their effects on direct markers of mitochondrial function in the trial.

The secondary objectives are: (1) to assess the impact of these interventions on glycemic and metabolic control outcomes including glycated hemoglobin (HbA1c), fasting plasma glucose (FPG), fasting insulin, homeostatic model assessment of insulin resistance (HOMA-IR), body weight, and body mass index (BMI); (2) to conduct a conditional NMA to compare and rank the relative efficacy of different intervention categories if the evidence network is sufficiently connected and clinically coherent; and (3) to explore potential sources of heterogeneity (e.g., intervention duration, baseline HbA1c levels) via prespecified subgroup and sensitivity analyses.

**Methods and analysis:**

Prepared per PRISMA-P, the review will follow PRISMA 2020 and PRISMA-NMA if applicable. Registered with PROSPERO (ID: CRD420251145356), searches will cover PubMed, Embase, Cochrane CENTRAL, Web of Science, and Scopus from inception to April 2026. Focused on T2DM, mitochondrial function/dysfunction, and interventions, age eligibility will be assessed during full-text screening. Primary analysis includes trials with participants aged 60+ or extractable data for this group; sensitivity analyses will include those with mean/median age ≥ 60. Two reviewers will handle screening, data extraction, and bias assessment using RoB 2.Analysis will focus on trials reporting mitochondrial function outcomes, categorized by biological domain, tissue source, and assay method. Glycemic control, geriatric health, and safety are secondary outcomes. Network meta-analysis will be conducted if a connected evidence network exists; otherwise, pairwise meta-analysis or narrative synthesis will be used. Random-effects models will be employed, with heterogeneity assessed via subgroup analysis and meta-regression. If significant heterogeneity persists, a Synthesis Without Meta-Analysis (SWiM) framework narrative review will be done. Evidence certainty will be appraised using GRADE and CINeMA.

**Ethics and dissemination:**

Ethics approval is not required for this systematic review as the systematic review will include only previously anonymised data from primary studies. Dissemination of the findings of the final systematic review will occur through publishing in a peer-reviewed journal. **PROSPERO registration number** CRD420251145356

## Introduction

Diabetes mellitus (DM), characterized by elevated blood glucose levels, continues to be a predominant cause of mortality and disability worldwide [1]. Type 2 diabetes mellitus (T2DM) represents the most prevalent form of the disease, exhibiting higher incidence rates and poorer prognoses among the elderly population [2]. T2DM is associated with an increased risk of complications, such as cardiovascular disease and retinopathy, which further exacerbate mortality and disability rates [3,4]. In older adults, the clinical manifestation of T2DM is often complicated by the presence of multiple comorbidities, polypharmacy, and geriatric syndromes, thereby elevating the risk of adverse events, including hypoglycemia and falls [5,6]. The progression of insulin resistance and the decline in β-cell compensatory capacity with age result in significant glycemic variability, complicating effective glycemic management. Current therapeutic strategies inadequately address the distinct physiological characteristics inherent in the older demographic. There is a notable deficiency in interventions that effectively balance glycemic control, quality of life, and long-term outcomes. Therefore, there is an urgent necessity for the development of individualized management strategies [7].

Mitochondrial dysfunction has been recognized as a common pathological mechanism underlying both T2DM and the aging process. Mitochondria play a crucial role in cellular energy metabolism; impairments in their function disrupt insulin signaling, leading to insulin resistance and pancreatic β-cell dysfunction [8,9]. Key factors contributing to the progression of T2DM and aging include diminished ATP production, elevated oxidative stress, and mitochondrial dysregulation [10]. With advancing age, there is a decline in mitochondrial biogenesis and the efficiency of the electron transport chain, accompanied by an increase in reactive oxygen species (ROS) production, resulting in a redox imbalance [11,12]. In individuals with diabetes, mitochondrial dysfunction is further aggravated, presenting as morphological abnormalities and reduced membrane potential, among other characteristics [13].

In elderly patients with T2DM, mitochondrial DNA mutations, impaired autophagy, and disrupted membrane potentials contribute to a cycle of metabolic stress-induced cellular damage [14]. Mitochondrial dysfunction has been associated with increased ROS)production through cardiolipin peroxidation, which in turn impairs ATP synthesis and mitochondrial autophagy [15]. Clinical studies have demonstrated that elderly individuals with T2DM exhibit reduced mitochondrial oxygen consumption and heightened oxidative stress levels. Furthermore, inadequate glycemic control has been shown to exacerbate mitochondrial dysfunction [16]. This functional decline promotes β-cell apoptosis, disrupts insulin secretion, and reduces both mitochondrial density and respiratory chain complex activity in peripheral tissues, thus advancing disease progression [17–19]. In the context of complications such as diabetic nephropathy and cardiomyopathy, hyperglycemia has been shown to enhance ROS production and accelerate mitochondrial fission, leading to multi-organ functional decline [20,21]. In conclusion, mitochondrial dysfunction represents a critical mechanism underlying increased glycemic variability and its associated complications.

Existing systematic reviews on T2DM interventions mostly focus on single modalities [22–24], include broad adult populations, and prioritize conventional metabolic outcomes over mitochondrial biomarkers [25,26]. No review has comprehensively compared diverse mitochondrial-targeted interventions specifically in older adults with T2DM, justifying the need for the present analysis.

Numerous pharmacological, nutritional, exercise-based, and dietary interventions have been shown to affect mitochondrial function in individuals with T2DM; however, their comparative effects in older adults remain inadequately characterized. While many of these interventions are not explicitly designed as mitochondrial-targeted therapies, they may nonetheless influence mitochondrial bioenergetics, oxidative stress, biogenesis, or mitophagy. Clinically, this comparison directly supports real-world clinical decision-making for older adults with T2DM, as clinicians routinely choose among pharmacological, nutritional, and lifestyle interventions. A conditional network meta-analysis, if feasible, will establish. Methodologically, all interventions converge on overlapping mitochondrial pathways, allowing holistic comparison of efficacy across intervention categories, with analyses only conducted when core transitivity assumptions are met.

This review will focus exclusively on randomized controlled trials where the intervention possesses a credible mitochondrial-related mechanism and reports at least one mitochondrial-related outcome. A network meta-analysis will be conducted only if the evidence network is connected and the assumptions of transitivity and clinical coherence are satisfied. In the absence of these conditions, either pairwise meta-analysis or a structured narrative synthesis will be employed. This document serves as the research protocol, and the completed PRISMA-P [27] checklist is provided in Additional File 1.

## Methods and analysis

### Study status

At the time of submission, the study has not yet generated results. Literature screening and data extraction have not been completed. Data collection is expected to be completed by June 2027, and the final results are anticipated by December 2027.

### Eligibility criteria

#### Study designs.

This protocol complies with the PRISMA-P guidelines [27]. The final review will be conducted in accordance with the PRISMA 2020 [28] statement and, where relevant, the PRISMA-NMA extension [29]. The protocol has been registered with PROSPERO. Recommendations from the Cochrane Handbook for Systematic Reviews of Interventions will be adhered to as applicable. Any modifications made during the review process will be transparently documented and updated on PROSPERO. The Eligibility criteria is shown in Table 1. Ethics approval is not required for this systematic review as the systematic review will include only previously anonymised data from primary studies.

**Table 1:** Eligibility criteria.

PICOS element	Inclusion criteria
Population	Older adults with T2DM; primary analysis restricted to trials enrolling participants aged ≥60 years or reporting extractable ≥60 subgroup data
Intervention	Pharmacological, supplement-based, exercise-based, or dietary interventions with plausible mitochondrial-related mechanisms and at least one mitochondrial outcome
Comparator	Placebo, usual care, no intervention, or active comparator
Primary outcomes	Mitochondrial-related outcomes, including mitochondrial respiration/oxidative phosphorylation, ATP production, mitochondrial membrane potential, mitochondrial DNA copy number, citrate synthase activity, mitochondrial biogenesis markers such as PGC-1α, NRF1/NRF2 and TFAM, mitochondrial dynamics markers such as MFN1/2, OPA1, DRP1 and FIS1, mitophagy markers such as PINK1, Parkin, LC3 and p62, and oxidative stress markers directly linked to mitochondrial dysfunction, including ROS, MDA, 8-OHdG, SOD, GPx and catalase.
Secondary outcomes	HbA1c, fasting glucose, HOMA-IR, frailty, sarcopenia, physical function, quality of life, adverse events
Study design	Randomized controlled trials, including parallel, crossover, and cluster RCTs

**Population:** This study will focus on older adults (aged ≥60 years) diagnosed with T2DM. T2DM will be defined according to established international guidelines, such as those from the American Diabetes Association (ADA) or the World Health Organization (WHO), based on criteria like HbA1c, fasting plasma glucose, or oral glucose tolerance test results as reported by the primary studies.The primary analysis will encompass studies where all participants are aged ≥60 years, as well as studies providing age-stratified data specifically for participants aged ≥60 years. Studies reporting a mean or median age of ≥60 years, but lacking an explicit age-restricted eligibility criterion, will be excluded from the primary analysis due to the potential inclusion of a significant proportion of younger participants. Such studies will be considered only in sensitivity or supportive analyses. Studies with a mean or median age of <60 years, without extractable data for an older-adult subgroup, will be excluded from the synthesis specific to the elderly population.

**Interventions:** Operational definition of mitochondrial-related interventions

To ensure reproducible and objective eligibility decisions, an intervention is defined as mitochondrial-related if it meets at least one of the following two predefined, non-mutually exclusive criteria:

(1) Mechanism-based: The intervention is a pharmacological/nutritional agent, exercise, or dietary strategy whose primary, well-established mechanism of action involves direct modulation of mitochondrial pathways (e.g., targeting respiratory chain complexes, regulating biogenesis via PGC-1α, or modifying mitophagy/dynamics), supported by prior peer-reviewed mechanistic or clinical literature.
(2) Outcome-based: The trial explicitly pre-specifies and measures at least one direct marker of mitochondrial function or structure as a primary or secondary endpoint, including but not limited to mitochondrial respiration, ATP production rate, mtDNA copy number, or expression of key regulators (PGC-1α, SIRT1, AMPK).

Eligibility decisions regarding mitochondrial relevance are made prior to outcome data extraction, with dual independent review and documented rationale for each classification. Interventions are further categorized as directly mitochondrial-targeted or indirectly mitochondrial-modulating during extraction.

Interventions that exhibit a plausible biological mechanism targeting mitochondria or are known to influence mitochondrial function will be considered. Eligible interventions will be categorized a priori into three primary groups:

(I) Mitochondria-targeted supplements or antioxidants, including coenzyme Q10, MitoQ, α-lipoic acid, nicotinamide riboside, nicotinamide mononucleotide, and other NAD+ precursors;
(II) Glucose-lowering pharmacological agents with documented effects on mitochondrial function, such as metformin, SGLT2 inhibitors, GLP-1 receptor agonists, and thiazolidinediones;
(III) Lifestyle, exercise, or dietary interventions with documented mitochondrial outcomes, including aerobic exercise, resistance training, high-intensity interval training, caloric restriction, time-restricted eating, or ketogenic diets.

Interventions will not qualify solely based on their ability to improve glycemic control; a mitochondrial-related rationale and at least one documented mitochondrial outcome are required. Broad or poorly defined interventions will be included only if their components, dosage or intensity, duration, and comparator are adequately described.

Stable background glucose-lowering and cardiometabolic therapies will be allowed. Studies in which background therapies are systematically altered during the intervention period or exhibit clear imbalances between groups will be evaluated for potential bias and indirectness. Detailed information on concomitant medications, particularly those known to influence mitochondrial function (e.g., metformin, statins), will be meticulously extracted. We will not exclude studies based on the use of these medications, as they often represent the standard of care. Instead, their potential confounding effects will be addressed through subgroup analysis (e.g., comparing studies with and without metformin as a background therapy) or meta-regression, contingent upon the availability of sufficient data. For the network meta-analysis (NMA), the distribution of these background therapies across comparisons will be examined during the transitivity assessment.

**Control group:** Within the framework of experimental design, it is imperative to consider various approaches, including the use of a placebo, the absence of intervention, the provision of routine care or standard treatment, or the implementation of an alternative active intervention, particularly in head-to-head trials. In the context of network meta-analysis, ensuring connectivity among interventions within the network is crucial.

**Outcome measures:** To be considered eligible, studies must report at least one outcome related to mitochondrial function. The primary focus of this review will be on outcomes associated with mitochondria, which will be categorized based on biological domain, tissue origin, and assay technique. The domains to be included are as follows:

Mitochondrial outcomes are prespecified into biologically distinct domains, with a hierarchical priority framework applied to ensure analytical consistency and clinical interpretability:

(1) Primary priority outcomes (core functional measures): Mitochondrial bioenergetics and respiration parameters, including ATP production, oxygen consumption rate, respiratory control ratio, oxidative phosphorylation markers, respiratory chain complex activity, and mitochondrial membrane potential. These direct functional endpoints are prioritized for all meta-analyses.
(2) Secondary priority outcomes (content and biogenesis markers): Mitochondrial biogenesis and mtDNA-related outcomes, including mtDNA copy number, citrate synthase activity, and expression of key regulatory factors (PGC-1α, TFAM, NRF1, NRF2). Oxidative stress markers (ROS, oxidative damage markers, antioxidant enzyme activity, lipid peroxidation markers) are included in this tier only when explicitly prespecified as mitochondrial-specific endpoints.
(3) Supportive priority outcomes (quality control markers): Outcomes related to mitochondrial dynamics and quality control, including (a) morphology and membrane structure markers (mitochondrial density, fission/fusion processes, ultrastructural markers); and (b) mitophagy-related proteins (PINK1, Parkin, LC3, p62, MFN1/2, OPA1, DRP1, FIS1).

The primary secondary metabolic outcomes to be assessed will encompass HbA1c levels, fasting plasma glucose, fasting insulin, HOMA-IR, insulin sensitivity indices, body weight, and BMI. Furthermore, additional secondary outcomes will include the incidence of adverse events, assessments of quality of life, measures of frailty, sarcopenia, grip strength, gait speed, physical function, cognitive outcomes, as well as cardiovascular and renal outcomes, contingent upon their availability in the data.

The primary endpoint of the study will be the conclusion of the intervention. In instances where multiple post-intervention time points are available. No study will be excluded solely on the basis of intervention duration, provided that the intervention period is clearly reported. Intervention duration will be extracted and, where sufficient studies are available, explored as a potential effect modifier using predefined categories: < 12 weeks, 12–24 weeks, and >24 weeks. The 12–24-week category was selected because many metabolic and lifestyle intervention trials in type 2 diabetes evaluate glycaemic and physiological changes within this timeframe.Longer-term follow-up data will be analyzed separately, contingent upon data availability.

Study design: This study is confined to randomized controlled trials (RCTs), encompassing parallel-group, crossover, and cluster-randomized designs. For crossover trials, only data from the first intervention period will be included in the primary analysis to prevent potential carryover effects from one intervention to the next. Excluded from this study are quasi-RCTs, non-randomized designs, single-arm before-and-after studies, and quasi-experimental designs. For a NMA to be feasible, a methodologically crucial requirement is the availability of a connected evidence network, which typically contains at least three different intervention nodes. This does not imply that each included RCT must be a three-arm trial.

### Search strategy

A thorough and systematic literature review will be performed utilizing the major electronic databases, encompassing the period from each database’s inception up to April 2026. The databases to be consulted include PubMed/MEDLINE, EMBASE (via Ovid), the Cochrane Central Register of Controlled Trials (CENTRAL), the Web of Science Core Collection, and Scopus. Where feasible, no language restrictions will be imposed.

To identify ongoing studies, unpublished literature, and grey literature, a comprehensive search strategy will be implemented. This will involve querying clinical trial registries such as ClinicalTrials.gov, the World Health Organization International Clinical Trials Registry Platform (ICTRP), and the ProQuest Global Dissertations Database. Furthermore, the EU Clinical Trials Register will be examined to identify ongoing or completed trials that have not yet been published. Manual searches will also be conducted on the reference lists of included studies and pertinent systematic reviews. Additionally, a grey literature search will be performed using Google Scholar, focusing on the first 200 results sorted by relevance.

The search strategy will be formulated by the principal investigators in collaboration with an experienced medical librarian, adhering to the Peer Review of Electronic Search Strategies (PRESS) guidelines. To ensure transparency and reproducibility, the search date, platforms, and complete search queries for each database will be meticulously documented. The primary search will operationalize the PICO framework into three searchable concepts: type 2 diabetes, mitochondrial function or dysfunction, and interventions or randomized/controlled trials.

Given the inconsistent indexing of age-related terms, age eligibility will be evaluated during the screening process. Additionally, a supplementary search focusing on age-related terms will be conducted to enhance the retrieval of studies explicitly involving older adults. The search strategy will be tailored for each database. Furthermore, reference lists of included studies and pertinent reviews will be manually screened.

In addition to the primary search strategy, which integrates terms related to T2DM, mitochondria, and RCTs/interventions, supplementary searches focused on specific interventions will be conducted. Specific drug and intervention names will be included in the supplementary search strategy, and the relevant intervention terms are detailed in the S2 File.

The complete search strategy for each database will be provided in S2 File.

### Data management and screening process

A standardized process involving dual independent review will be used for all stages of study selection, data extraction, and risk of bias assessment. Covidence software will facilitate this process. Two reviewers will independently perform each task; any disagreements will be resolved through discussion or, if consensus cannot be reached, by a third senior reviewer.

The process of data collection and extraction will adhere to standardized systematic review methodologies. The management of screening and data extraction will be facilitated through the use of Covidence or comparable software.

(I) **Literature Screening:** The process commences with an initial screening of titles and abstracts. During the full-text review, the two reviewers independently evaluate the studies, with any disagreements being resolved by a third senior reviewer. The number of excluded articles and the reasons for their exclusion at each stage are meticulously documented, and a PRISMA flowchart is subsequently generated.
(II) **Design of the Data Extraction Form:** A standardized pre-trial data extraction form will be developed using the Covidence platform. This form will encompass general study characteristics, details of the intervention, and parameters related to mitochondrial function. General information will include publication ID, authors, year, country, study design, sample size, and population characteristics such as age range, age standard deviation, proportion of participants aged ≥60 or ≥65, whether age-stratified data were reported, the age cut-off used, whether older subgroup data were obtained from authors, gender, disease duration, and HbA1c levels, among other variables.

The extracted details encompass the name of the intervention, its dosage, frequency, and duration; its administration as adjunct therapy; the type of control group utilized; the time points for outcome measurement; baseline values; the dropout rate; and the appropriateness for network meta-analysis, including intervention nodes and the number of arms.

A prespecified intervention-node framework will be developed before screening is completed. The framework will record the intervention name, dose or intensity, duration, proposed mitochondrial mechanism, source supporting the mechanism, and assigned node.

(III) **Data Extraction:** The primary outcomes of interest include parameters of mitochondrial function, while secondary outcomes encompass metabolic markers such as HOMA-IR and HbA1c, as well as age-specific clinical outcomes, including frailty, sarcopenia, cognitive function, and cardiovascular and renal outcomes. Details of the interventions, including dosage, frequency, duration, co-interventions, adherence, and characteristics of comparators, will also be extracted. The techniques used for mitochondrial detection, such as Seahorse respirometry, Western blotting, and PCR, along with the types of samples analyzed, including skeletal muscle, blood, and adipose tissue, must be clearly specified. In cases where outcomes are reported in varying units, conversions to a standardized unit will be performed when appropriate. Data presented solely in graphical form will be extracted using validated digital extraction software, and the accuracy of this extraction will be independently verified by two reviewers.
(IV) **Management of Special Circumstances and Quality Assurance:** In studies involving multiple groups, data will be extracted from all pertinent groups, taking into account intergroup correlations. In instances where multiple reports are available for the same study, the meta-analysis will prioritize the most recent or the most comprehensive version. Any disagreements arising during the data extraction process will be resolved through consensus, with the option of third-party arbitration if necessary.

### Risk of bias(RoB) assessment

This study will employ a hierarchical, stepwise framework for bias assessment. The outcomes of the bias assessment will guide sensitivity analyses, such as reanalysis excluding studies identified as high-risk. The quality of evidence for the outcomes will be evaluated using the GRADE [30,31] system.

The risk of bias in individual randomized trials will be evaluated utilizing the Cochrane Risk of Bias 2.0 (RoB 2.0) tool [32]. For parallel-group RCTs, the standard RoB 2.0 tool will be employed, while the appropriate RoB 2.0 variants will be applied to cluster-randomized or crossover trials, as applicable. The RoB 2.0 tool encompasses five domains: bias arising from the randomization process, bias due to deviations from intended interventions, bias due to missing outcome data, bias in the measurement of the outcome, and bias in the selection of the reported result. Each study outcome will be assessed and categorized as having a low, some concerns, or high risk of bias.

These risk of bias assessments will inform sensitivity analyses and the evaluation of the certainty of evidence. In the context of network meta-analysis, risk of bias will be integrated into the CINeMA [33] framework, rather than being assessed using a distinct NMA risk-of-bias tool.

### Data synthesis and statistical analysis

This study has been pre-registered with PROSPERO (registration number: CRD420251145356). All procedures adhere to the pre-registered protocol. Any deviations from this protocol will be reported in the results section, accompanied by justifications and an analysis of potential implications. All modifications will be systematically documented with traceable records.

#### General methodological framework.

This section defines the overarching principles for data synthesis.

**Effect Measures:** For continuous outcomes, mean difference (MD) will be used when data are on the same scale, and standardized mean difference (SMD) when data are on different scales; for dichotomous outcomes, odds ratios (OR) or risk ratios (RR) will be used. All estimates will be presented with 95% confidence intervals (CI). For specific indicators, such as peripheral blood mitochondrial DNA copy number (mtDNAcn), the effect measure will be chosen according to the measurement method most frequently used in the included studies to ensure comparability. Because mitochondrial outcomes are expected to vary by tissue source and measurement platform, analyses will be stratified by biomarker type, biological sample, and assay method; outcomes measuring fundamentally different constructs will not be pooled unless they can reasonably be interpreted as reflecting the same underlying domain. SMD values will be interpreted using conventional thresholds (0.2 = small, 0.5 = moderate, 0.8 = large) and, where possible, back-transformed to the original units using pooled baseline standard deviations.

**Analysis Model:** All meta-analyses will primarily use a random-effects model, with between-study variance (τ^2^) estimated by restricted maximum likelihood (REML). The Hartung-Knapp adjustment will be applied where appropriate, particularly when the number of studies per comparison is small; DerSimonian-Laird estimates may be explored in sensitivity analyses but will not be used as the primary estimator.

Statistical significance will be assessed using two-sided P < 0.05. A random-effects meta-analysis will be performed when at least three studies are available and clinical heterogeneity is adequately controlled; pooled estimates will be avoided when heterogeneity is clinically significant, as indicated by opposing effect directions or I^2^ > 75%. Further details are provided in the Heterogeneity Assessment and Management Strategies section.

#### Heterogeneity assessment and management strategies.

Prior to data synthesis, heterogeneity will be systematically assessed across three dimensions:

Clinical heterogeneity is indicated. A qualitative comparison will be used to evaluate the differences in baseline characteristics (age range, disease severity), intervention protocols (type, dose, duration), and outcome measurement methods of the included studies. Significant heterogeneity will be addressed through subgroup analysis.

Methodological heterogeneity will be addressed through a comprehensive evaluation aimed at identifying variations in study design. The risk of bias will be systematically assessed utilizing the RoB 2 tools to uphold methodological rigor. Data collection methods will undergo thorough examination to confirm the reliability of the sources. All eligible studies will be included in the primary synthesis regardless of risk-of-bias judgement. Sensitivity analyses will be conducted by excluding studies judged to be at high risk of bias. Risk-of-bias assessments will also inform the certainty of evidence using GRADE or CINeMA.

Statistical Heterogeneity: The assessment of data heterogeneity will be conducted utilizing the Cochrane Q statistic, where a P-value of less than 0.10 signifies heterogeneity, alongside the I^2^ statistic and τ^2^, estimated via the REML method. The interpretation of I^2^ values will adhere to the guidelines specified in the Cochrane Handbook version 6.4, categorizing data as follows: 0–40% (low heterogeneity), 30–60% (moderate heterogeneity), 50–90% (substantial heterogeneity), and 75–100% (high heterogeneity). Priority will be given to the analysis of prespecified covariates, such as age and HbA1c, to mitigate the risk of multiple testing bias. This study will adopt a multidimensional approach to evaluate heterogeneity, integrating the degree of clinical heterogeneity, consistency in effect direction, and a τ^2^ threshold greater than 0.5. Furthermore, clinical relevance will be assessed through the use of prediction intervals.

If I^2^ > 50% or Q test p < 0.10, we will first re-check extracted data and examine study-level characteristics (e.g., differences in outcome measurement, population, or design) to identify potential sources. Subgroup analyses will be planned only when there is a plausible biological or clinical rationale, not simply because of a high I^2^ value.

### Effects of mitochondria-related interventions

#### Network Meta-analysis (Conditional).

NMA is an extension of pairwise meta-analysis, and it is conducted only when the following strict criteria are met simultaneously:

(I) Network Connectivity: NMA will be performed contingent upon the treatment network being connected and comprising a minimum of three intervention nodes. The existence of closed loops within the network will allow for a formal evaluation of local incoherence. In the absence of closed loops, incoherence will be assessed qualitatively, necessitating a cautious interpretation of the certainty of evidence. Intervention nodes are categorized by intervention type (e.g., exercise, coenzyme Q10, metformin, etc.). Interventions exhibiting excessive heterogeneity will not be aggregated into a single node. The network structure for each outcome will be validated through visualization.
(II) Sufficient Evidence Base:NMA will be undertaken contingent upon the availability of a sufficient evidence base.

For the purpose of this protocol, a sufficient evidence base for NMA will be defined as a connected network with at least three intervention nodes and at least one direct study informing each connection. We will also examine whether the number of studies and participants is adequate to support stable estimation. In instances where data are sparse, results will be interpreted with caution, with analysis restricted to pairwise meta-analysis or narrative synthesis as appropriate.

(III) The validity of the assumptions is as follows:

Transitivity: Transitivity will be evaluated by analyzing the distribution of predefined effect modifiers across treatment comparisons, such as age, duration of diabetes, baseline HbA1c levels, body mass index (BMI), concurrent medications, comorbidities, duration of intervention, and methods of outcome measurement. A substantial imbalance will be identified when differences are clinically significant and have the potential to alter treatment effects. Given the variability in intervention measures, we will conduct a thorough examination of the distribution of potential effect modifiers across various pairwise comparisons. Key modifiers include baseline age, duration of diabetes, baseline glycated hemoglobin (HbA1c) levels, and concurrent standard treatment medications. In the presence of significant conceptual heterogeneity between pharmacological and lifestyle intervention trials, we will consider the development of independent sub-networks or the application of network meta-analysis to adjust for these modifiers.

Consistency: NMA will be performed provided that the treatment network is interconnected and comprises a minimum of three intervention nodes. The existence of closed loops within the network will enable a formal evaluation of local inconsistency. In the absence of such loops, inconsistency testing cannot be conducted, necessitating a qualitative assessment of transitivity.

If the aforementioned conditions are not satisfied, a pairwise meta-analysis will be undertaken in conjunction with a narrative synthesis. Subsequently, effect directions will be illustrated using an albatross plot, and an informal ranking will be conducted based on effect sizes and precision. The CINeMA rating will form the basis for the GRADE assessment, and the evidence grade will be downgraded by one level in the presence of significant inconsistency issues. Planned analytical decision framework is shown in Fig 1.

**Fig 1:**
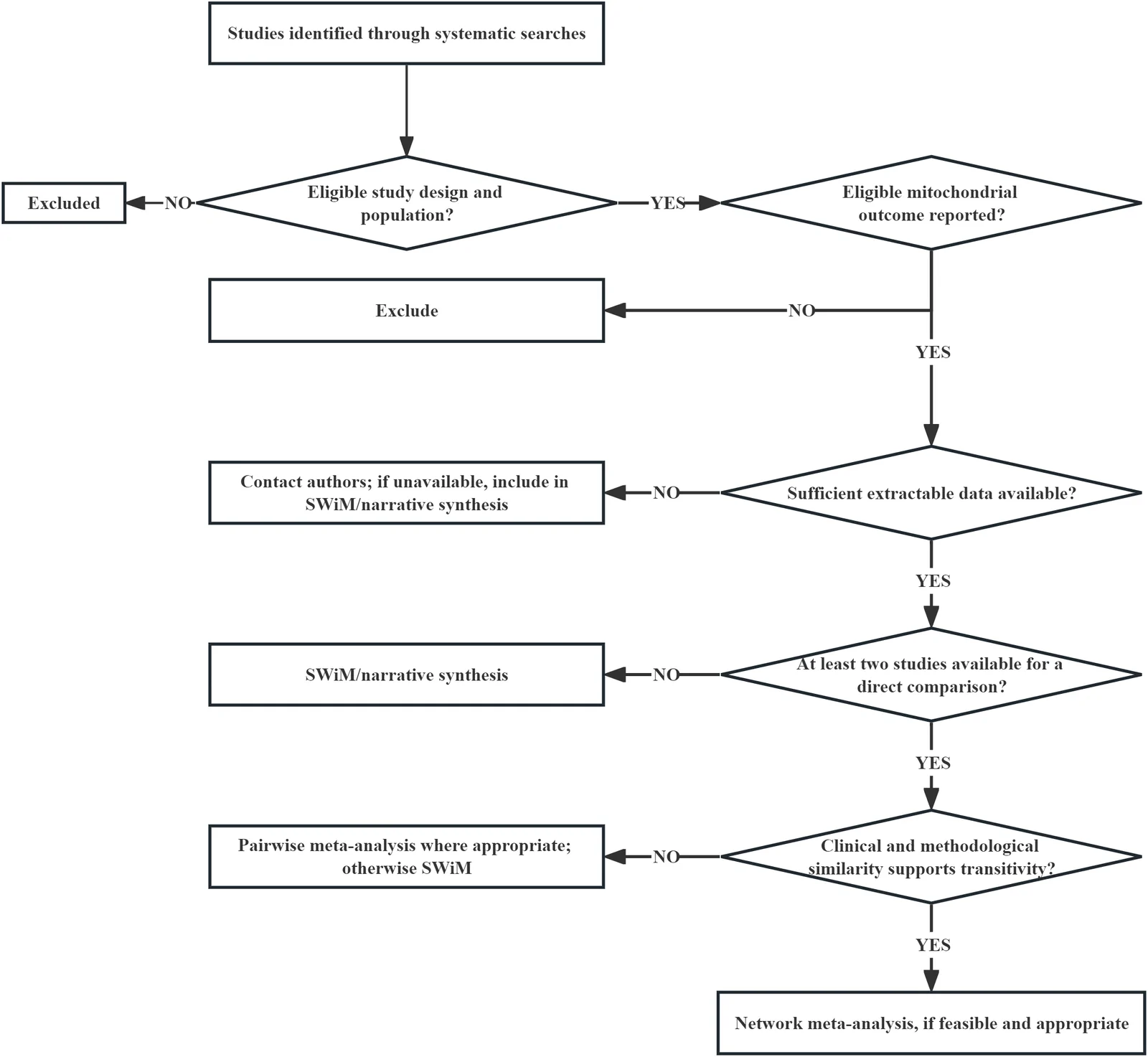
Planned analytical decision framework. NMA will be undertaken only when a connected treatment network is available for a given outcome and the transitivity assumption is considered clinically and methodologically plausible. Statistical inconsistency will be assessed after NMA, where feasible. If quantitative synthesis is not appropriate, findings will be synthesised following SWiM guidance.RCT, randomized controlled trial; NMA, network meta-analysis; SWiM, synthesis without meta-analysis.

**Effect measures and data integration strategy:** The model selection process is outlined as follows: The primary NMA will employ a frequentist random-effects model, executed in R using the netmeta package. Bayesian random-effects models may be utilized for sensitivity analyses. Consistent node definitions and outcome domains will be applied across both frequentist and Bayesian analyses to ensure comparability.

Effect sizes will follow the general principles described in the General Methodological Framework (MD or SMD for continuous outcomes; OR for dichotomous outcomes) and will be presented with 95% confidence intervals in the frequentist analysis or 95% credibility intervals in the Bayesian analysis.

**Intervention node:** The definition and classification of these intervention nodes will be established a priori, before any data synthesis or observation of study results, to prevent bias.

Interventions will be categorized into clinically meaningful nodes based on their mechanisms of action and modalities of intervention. The classification of nodes will be determined prior to data analysis and subsequently reviewed by clinical experts. Individual interventions will be considered as distinct nodes when sufficient data are available. However, interventions with similar mechanisms may be grouped into broader nodes only if deemed clinically comparable by at least two clinical experts, and when separate-node analysis is not feasible. A comprehensive network encompassing all pharmacological, supplement-based, exercise-based, and dietary interventions will be constructed only if the transitivity assumption is deemed clinically plausible. In cases where there is significant conceptual or clinical heterogeneity across intervention classes, separate networks will be developed for pharmacological interventions, mitochondrial-targeted supplements, and lifestyle interventions. If a coherent connected network cannot be established for a particular outcome, a NMA will not be conducted for that outcome(Table 2.).

**Table 2:** Prespecified intervention nodes for potential network meta-analysis.

First-level category (domain)	Prespecified node	Grouping rule (basis for a separate node)	Representative examples
Glucose-lowering pharmacological agents	Metformin	Each drug forms its own node; separate dose-based subgroups only if ≥3 studies per stratum	Metformin
	SGLT2 inhibitors	Drugs of the same class are combined into one node; split into individual drugs only if ≥3 studies per drug	Empagliflozin, dapagliflozin
	GLP-1 receptor agonists	Drugs of the same class are combined into one node; split into individual drugs only if ≥3 studies per drug	Liraglutide, semaglutide
	Thiazolidinediones	Drugs of the same class are combined into one node	Pioglitazone
Mitochondria-targeted supplements/antioxidants	CoQ10	Each drug forms its own node	Ubiquinone, ubiquinol
	MitoQ	Each drug forms its own node	MitoQ
	α-lipoic acid	Each drug forms its own node	α-lipoic acid
	NAD+ precursors	Drugs of the same class are combined into one node (NR and NMN); split only if ≥3 studies per drug	Nicotinamide riboside (NR), nicotinamide mononucleotide (NMN)
Exercise/lifestyle interventions	Aerobic exercise	Each exercise type forms its own node	Continuous moderate-intensity training
	Resistance training	Each exercise type forms its own node	Progressive resistance training
	Combined exercise	Combined exercise (aerobic + resistance) forms its own node	Combined training
	High-intensity interval training (HIIT)	Each exercise type forms its own node	HIIT
	Dietary restriction	Each diet type forms its own node	Caloric restriction, time-restricted eating, ketogenic diet
	Multicomponent lifestyle intervention	Multi-component programme (e.g., exercise + diet + behavioural support) forms its own node	Multicomponent programme

(I) **First-level category:** domains used to organise the evidence base, they are not NMA nodes:(a) Glucose-lowering pharmacological interventions with reported mitochondrial effects, (b) Mitochondria-targeted supplements or antioxidants, (c) Lifestyle and exercise interventions affecting mitochondrial function
(II) **Prespecified node:** the intervention categories that will enter the network meta-analysis. Each eligible intervention will be assigned to a node on the basis of its active component(s), proposed mitochondrial mechanism, and modality of delivery, and this assignment, together with the source supporting the proposed mechanism, will be recorded in the prespecified intervention-node framework (see Data Management and Screening Process). An intervention will be retained as a separate node only when it is supported by at least three independent studies; otherwise, clinically exchangeable interventions will be combined into a single node. Two interventions will be grouped into the same node only when they are considered clinically exchangeable with respect to mechanism of action, delivery modality, and expected effect on the relevant mitochondrial outcome domain. Broad categories (e.g., “nutritional interventions”) will not constitute a single node, and interventions exhibiting excessive clinical or methodological heterogeneity will not be aggregated. The provisional list of candidate nodes, their grouping rules, and representative examples is provided in Table 2. If a connected network cannot be established for a node, that node will be excluded from the network meta-analysis and analysed using pairwise meta-analysis or narrative synthesis. Any modification of node definitions required by unanticipated variation in the evidence base will be documented and reported transparently, with the rationale and timing of the change.

**Outcome-specific networks.** In line with the a priori definition of intervention nodes, separate networks will be constructed for distinct mitochondrial outcome domains, namely, mitochondrial biogenesis; oxidative phosphorylation or respiratory function; ATP-related outcomes; mitochondrial DNA measures; mitochondrial dynamics or mitophagy; and mitochondrial redox or oxidative-stress measures. Outcomes from different domains will not be combined within the same network. Within a domain, quantitative synthesis will be considered only when the underlying construct and measurement approach are sufficiently compatible; otherwise, results will be summarised narratively. If a connected network cannot be established for a given outcome domain, that outcome will be excluded from the network meta-analysis and handled through pairwise meta-analysis or narrative synthesis.

**Result presentation and evidence grading**

(I) The presentation of effect size is as follows: The pooled effects of interventions relative to a common control (e.g., placebo) will be presented using a forest plot, supplemented by a pairwise ranking table.
(II) Treatment ranking: The relative efficacy of the interventions will be assessed using the area under the cumulative ranking curve (SUCRA) and mean rank. The 95% confidence intervals (CIs) and credible intervals (CrIs) will be reported, and their probabilistic nature will be specified.
(III) The degree of certainty of the evidence is as follows: The CINeMA framework will be utilized to assess the quality of evidence, incorporating the following quantitative downgrade thresholds: Downgrading decisions will be informed by the magnitude and potential impact of each limitation, rather than relying solely on fixed numerical thresholds. Any quantitative thresholds employed to support judgments will be predetermined and interpreted in the context of clinical relevance and the significance of the outcome. Additional evaluation dimensions include indirectness, imprecision, and heterogeneity (refer to Section Heterogeneity Assessment and Management Strategies for detailed methodologies). Treatment rankings will be interpreted with caution and will not serve as the sole basis for conclusions. Rankings will be presented alongside effect estimates, confidence or credible intervals, clinical relevance, and the certainty of evidence. No definitive clinical recommendation will be made based solely on SUCRA or mean rank.

#### Pairwise meta-analysis.

**Effect measures and data integration strategy**

(I) Continuous outcomes: Continuous mitochondrial outcomes will be analyzed using the effect measures described in the General Methodological Framework. Indicators of glycemic control, including HbA1c, fasting blood glucose, and HOMA-IR, will primarily be reported as changes from baseline, calculated as the difference between the post-intervention mean and the baseline mean, along with the standard deviation. In cases where baseline data are unavailable, post-intervention endpoints will be utilized. The statistical analysis will involve assessing differences between the intervention and control groups. Effect sizes will be reported with 95% CIs.
(II) Binary Outcomes: Adverse events associated with treatment will be quantified using relative risk (RR) to evaluate safety differences. In the case of rare events, suitable statistical methods, such as Peto odds ratios or generalized linear mixed models, will be employed in sensitivity analyses, contingent upon the distribution of events and group balance. The intention-to-treat (ITT) analysis will be prioritized over the per-protocol analysis, with sensitivity analyses performed to examine any observed differences.

**Meta-analysis model selection:** In instances where only a single study is available, a structured narrative review will be performed, ensuring comprehensive documentation of all findings. The heterogeneity of the data will be evaluated through the integration of the I^2^ statistic, Cochran’s Q test, and τ^2^ values (refer to Section Heterogeneity Assessment and Management Strategies for detailed methodologies), without dependence on a singular I^2^ threshold. To detect significant heterogeneity, the following approach will be utilized: initially, outliers will be identified via forest plots and clinical characteristics; subsequently, pre-specified subgroup analyses and meta-regression will be undertaken. When the number of pre-specified subgroups is five or more, the Bonferroni correction will be applied to adjust the threshold for statistical significance (P-value).Should significant unexplained heterogeneity preclude pooled analysis, a narrative review adhering to the Synthesis Without Meta-Analysis (SWiM) [34]framework will be implemented.

**Subgroup analysis (each subgroup included ≥3 studies):** All subgroup analyses will be conducted only if at least three independent studies are available per stratum, and all findings will be considered exploratory and interpreted with appropriate caution.

(I) Age group: Stratified into 60–74 years (“young-old”) versus ≥75 years (“old-old”), consistent with standard geriatric clinical classifications. If trials report age using alternative cut-offs, we will harmonize categories as closely as possible (e.g., by assignment based on reported mean/median age), with mixed-age studies excluded if disaggregated data are unavailable.
(II) Intervention category: The following drugs have been identified as effective in targeting mitochondria: MitoQ, coenzyme Q10, metformin, SGLT2 inhibitors, GLP-1 receptor agonists, and pioglitazone.

In addition, the following non-pharmacological interventions have been identified as effective in targeting mitochondria:

a. Different types of exercise, including aerobic exercise, resistance training, and high-intensity interval training
b. Dietary interventions, such as calorie restriction and the ketogenic diet
c. Mitochondrial nutrient supplements (NMN, NR, α-lipoic acid). Finer within-class analyses will only be conducted post-hoc if data density permits.

(III) Intervention duration: Initially categorized as short-term (≤12 weeks) versus long-term (>12 weeks). If ≥3 studies are available per stratum, a further split into 12–24 weeks and >24 weeks may be explored as a post-hoc sensitivity analysis; narrow duration categories will not be used for primary subgroup comparisons.
(IV) Baseline glycemic control: Stratified by mean/median baseline HbA1c using a clinically relevant threshold of <7.5% versus ≥7.5%, consistent with guideline-recommended targets for older adults with T2DM [35]. Studies using incompatible categorization will be excluded from this analysis unless missing disaggregated data can be obtained from study authors. Multiple HbA1c thresholds will not be employed for primary analyses to avoid data fragmentation.

**Sensitivity analysis strategy:** Studies identified as having a high risk of bias, as per the RoB 2 criteria (“high risk”), should be excluded from the analysis. The subsequent phase of the research will involve validating the fixed-effects model. The influence of individual studies should be evaluated through a stepwise exclusion method. It is advisable to compare per-protocol data with intention-to-treat analysis, if such data is available. Studies characterized by a high rate of missing data, specifically those with a follow-up rate of less than 80%, have been excluded from the analysis.

### Handling of multi-arm trials

Within-study correlations will be addressed using the adjusted variance approach. Pairwise analyses will include only the pertinent groups to prevent double-counting, whereas NMA will include all groups and model between-group correlations.

### Publication bias and evidence certainty

When a minimum of ten studies are incorporated for a specific outcome measure, a funnel plot will be employed for visualization purposes, alongside Egger’s regression test, where a P-value of less than 0.1 denotes significant asymmetry. For binary outcomes, the Harbord test will additionally be utilized. Should bias be identified, the effect size will be recalibrated using the trim-and-fill method. In instances where fewer than ten studies are available, a visual assessment will be performed, supplemented by a detailed documentation of the study’s inherent limitations.

In the event that a minimum of 30% of the included studies are assessed as having a high RoB 2, the overall grade will be reduced by one level. Similarly, a downgrade by one level will occur if the 95% CI intersects the null line and the total sample size is fewer than 1,000. Additional factors that may lead to a downgrade include inconsistency, indirectness (such as surrogate outcomes or short-term follow-up), and publication bias. Given that only RCTs will be included, the evidence will initially be rated as high certainty. However, it may be downgraded based on risk of bias, inconsistency, indirectness, imprecision, or publication bias. The final grade will be categorized as high, moderate, low, or very low, with comprehensive documentation provided for the rationale behind each grading decision.

### Data missing value handling strategy

Standard Deviation Interpolation: Following the protocols delineated in the 6th Edition of the Cochrane Handbook, standard deviations (SDs) will be extracted from studies employing identical measurement scales and conducted at concurrent time points for the purpose of interpolation. In instances where interpolation is not viable, the SD/mean ratio will be estimated using the median ratio reported in systematic reviews pertinent to the same field. The validity of all interpolation methodologies will be assessed through sensitivity analyses, which will include the exclusion of studies where the proportion of interpolated data surpasses 20%.

In instances where baseline change data are absent, interpolation will be conducted utilizing a correlation coefficient of 0.5, ascertained from a meta-analysis of longitudinal studies focusing on mitochondrial biomarkers. The robustness of this interpolation approach will be assessed through sensitivity analyses, wherein the correlation coefficient (r) will be evaluated at values of 0.2 and 0.8. Missing standard deviations will be estimated from available standard errors, confidence intervals, P values, t statistics, or interquartile ranges whenever feasible. In cases where these data are not accessible, standard deviations may be imputed following the methodologies recommended by the Cochrane Handbook. The effect of such imputations will be further scrutinized through sensitivity analyses.

## Dissemination

Dissemination of the findings of the final systematic review will occur through publishing in a peer-reviewed journal.

## Supporting information

S1 File This is the PRISMA-P-checklist for protocol. (PDF)

S2 File Search strategy. (PDF)
