# Supplementary material for: Interventions with mitochondrial-related effects in older adults with type 2 diabetes: Protocol for a systematic review with conditional network meta-analysis
Source: PLoS One. 2026 Oct 16;21(10):e0360683. doi: 10.1371/journal.pone.0360683 (PMC13674747; doi:10.1371/journal.pone.0360683)
Supplement: Supplementary file 2 [file pone.0360683.s002.pdf]

Records retrieved from supplementary searches will be deduplicated against the primary search results. All records, regardless of whether they are identified through the primary or supplementary searches, will be screened using the same predefined eligibility criteria. Studies will be eligible only if they include older adults with T2DM or report extractable data for this population and assess at least one mitochondrial-related outcome. The supplementary searches are intended to increase retrieval sensitivity and will not broaden the review eligibility criteria.

The search strategy will be developed by the primary investigators in consultation with an experienced medical librarian, adhering to the Peer Review of Electronic Search Strategies (PRESS) guidelines. To ensure transparency and reproducibility, the search date, platforms, and complete search queries for each database will be fully documented.

Controlled vocabulary includes: The utilisation of subject headings specific to the database in question is paramount, with examples including the Medical Subject Headings (MeSH) provided by PubMed and the Emtree employed by Embase. This process is instrumental in ensuring the retrieval of literature that has been correctly indexed.

Free-text Terms: A search for synonyms, abbreviations, alternative spellings, and related terms is to be conducted in the Title, Abstract, and Keywords fields. The utilisation of truncation symbols (*), in conjunction with wildcards (e.g. ? or $), is recommended in order to expand the scope.

Concept 1: T2DM

MeSH/Emtree: Diabetes Mellitus, Type 2

Free-text: type 2 diabet*, T2DM, NIDDM, non-insulin dependent diabet*, adult onset diabet*, maturity onset diabet*

Concept 2: Mitochondrial Dysfunction

MeSH/Emtree: Mitochondria, Mitochondrial Diseases, Mitochondrial Myopathies, Energy Metabolism, Oxidative Phosphorylation, Oxidative Stress, Mitochondrial DNA

Free-text:

Core terms: mitochondri* (covers mitochondrion, mitochondria, mitochondrial)

Function/Dysfunction: dysfunction*, impairment*, abnormality*, defect*, insufficiency*

Biological processes: biogenesis, mitophagy, autophagy, dynamics, fission, fusion, mitofusin*, OPA1, DRP1

Energy Metabolism/Respiratory Chain: energy metabolism, bioenergetic*, respiration, oxidative phosphorylation, OXPHOS, electron transport chain, ETC, respiratory chain, complex I, complex II, complex III, complex IV, complex V, ATP synthase

Oxidative Stress: oxidative stress, reactive oxygen species, ROS, antioxidant*, superoxide, lipid peroxidation

DNA/Genes/Proteins: mitochondrial DNA, mtDNA, copy number, deletion*, mutation*, PGC-1alpha, PPARGC1A, TFAM, NRF1, NRF2, SIRT1, SIRT3, PINK1, Parkin, UCP* (Uncoupling Protein)

Functional assays: ATP production, oxygen consumption, membrane potential

Concept 3: Intervention or randomized controlled trial：

MeSH/Emtree: Clinical Trial, Randomized Controlled Trial, Intervention Studies

Free-text: intervention*, treatment*, therapy, exercise, diet, lifestyle, pharmacological, drug*, supplement*

supplementary search 1: Elderly

MeSH/Emtree: Aged, Frail Elderly, Aged, 80 and over
Free-text: elderly, aged, older adult*, older people, geriatric*, senior*, old age

PubMed search strategy

#1 "Diabetes Mellitus, Type 2"[Mesh] OR "type 2 diabetes"[tiab] OR "type 2 diabetic"[tiab] OR T2DM[tiab] OR T2D[tiab] OR NIDDM[tiab] OR "non-insulin-dependent diabetes"[tiab] OR "adult-onset diabetes"[tiab]
#2 "Mitochondria"[Mesh] OR "Mitochondrial Proteins"[Mesh] OR mitochondria*[tiab] OR mitochondrial[tiab] OR "mitochondrial dysfunction"[tiab] OR "mitochondrial function"[tiab] OR "mitochondrial respiration"[tiab] OR "oxidative phosphorylation"[tiab] OR OXPHOS[tiab] OR "electron transport chain"[tiab] OR "respiratory chain"[tiab] OR "ATP production"[tiab] OR "ATP synthesis"[tiab] OR "mitochondrial membrane potential"[tiab] OR "mitochondrial DNA"[tiab] OR mtDNA[tiab] OR "mtDNA copy number"[tiab] OR "mitochondrial biogenesis"[tiab] OR "PGC-1 alpha"[tiab] OR "PGC-1α"[tiab] OR TFAM[tiab] OR "citrate synthase"[tiab] OR "complex I"[tiab] OR "complex II"[tiab] OR "complex III"[tiab] OR "complex IV"[tiab] OR "complex V"[tiab] OR "mitochondrial reactive oxygen species"[tiab] OR "mitochondrial ROS"[tiab]
#3 "Aged"[Mesh] OR "Aged, 80 and over"[Mesh] OR elderly[tiab] OR aged[tiab] OR aging[tiab] OR ageing[tiab] OR geriatric*[tiab] OR "older adult*"[tiab] OR "older people"[tiab] OR "older patient*"[tiab] OR "older population*"[tiab] OR senior*[tiab]
#4 "Randomized Controlled Trial"[Publication Type] OR "Controlled Clinical Trial"[Publication Type] OR randomized[tiab] OR randomised[tiab] OR randomly[tiab] OR placebo[tiab] OR trial[tiab] OR "clinical trial"[tiab] OR intervention*[tiab] OR treatment*[tiab] OR therap*[tiab] OR exercise[tiab] OR training[tiab] OR diet*[tiab] OR nutrition*[tiab] OR lifestyle[tiab] OR "physical activity"[tiab] OR pharmacological[tiab] OR drug*[tiab] OR medication*[tiab] OR supplement*[tiab]
#5 animals[mh] NOT humans[mh]
Final search:
(#1 AND #2 AND #4) NOT #5
Additional age retrieval：(#1 AND #2 AND #3 AND #4) NOT #5

Embase

#1 'type 2 diabetes mellitus'/exp OR 'type 2 diabetes':ti,ab OR 'type 2 diabetic':ti,ab OR t2dm:ti,ab OR t2d:ti,ab OR niddm:ti,ab OR 'non insulin dependent diabetes':ti,ab OR 'adult onset diabetes':ti,ab
#2 'mitochondrion'/exp OR 'mitochondrial protein'/exp OR mitochondria*:ti,ab OR mitochondrial:ti,ab OR 'mitochondrial dysfunction':ti,ab OR 'mitochondrial function':ti,ab OR 'mitochondrial respiration':ti,ab OR 'oxidative phosphorylation':ti,ab OR oxphos:ti,ab OR 'electron transport chain':ti,ab OR 'respiratory chain':ti,ab OR 'ATP production':ti,ab OR 'ATP synthesis':ti,ab OR 'mitochondrial membrane potential':ti,ab OR 'mitochondrial DNA':ti,ab OR mtdna:ti,ab OR 'mitochondrial biogenesis':ti,ab OR 'PGC-1 alpha':ti,ab OR 'PGC-1α':ti,ab OR tfam:ti,ab OR 'citrate synthase':ti,ab OR 'complex I':ti,ab OR 'complex II':ti,ab OR 'complex III':ti,ab OR 'complex IV':ti,ab OR 'complex V':ti,ab OR 'mitochondrial reactive oxygen species':ti,ab OR 'mitochondrial ROS':ti,ab
#3 'aged'/exp OR elderly:ti,ab OR aged:ti,ab OR ageing:ti,ab OR aging:ti,ab OR geriatric*:ti,ab OR 'older adult*':ti,ab OR 'older people':ti,ab OR 'older patient*':ti,ab OR senior*:ti,ab

#4 'randomized controlled trial'/exp OR 'clinical trial'/exp OR random*:ti,ab OR placebo*:ti,ab OR trial:ti,ab OR intervention*:ti,ab OR treatment*:ti,ab OR therap*:ti,ab OR exercise:ti,ab OR training:ti,ab OR diet*:ti,ab OR nutrition*:ti,ab OR lifestyle:ti,ab OR 'physical activity':ti,ab OR pharmacological:ti,ab OR drug*:ti,ab OR medication*:ti,ab OR supplement*:ti,ab
#5 'animal'/exp NOT 'human'/exp
Final search:
#1 AND #2 AND #4 NOT #5
Additional age retrieval：#1 AND #2 AND #3 AND #4 NOT #5

Cochrane CENTRAL

("type 2 diabetes" OR T2DM OR T2D OR NIDDM OR "non-insulin-dependent diabetes") AND(mitochondria* OR mitochondrial OR "mitochondrial dysfunction" OR "mitochondrial function" OR "mitochondrial respiration" OR "oxidative phosphorylation" OR OXPHOS OR "electron transport chain" OR "ATP production" OR "mitochondrial membrane potential" OR mtDNA OR "mitochondrial biogenesis" OR "PGC-1 alpha" OR TFAM OR "citrate synthase" OR "mitochondrial ROS")
Additional age retrieval： AND(elderly OR aged OR geriatric* OR "older adult*" OR "older patient*")

Web of Science

TS = (("type 2 diabetes" OR "type 2 diabetic" OR T2DM OR T2D OR NIDDM OR "non-insulin-dependent diabetes")AND(mitochondria* OR mitochondrial OR "mitochondrial dysfunction" OR "mitochondrial function" OR "mitochondrial respiration" OR "oxidative phosphorylation" OR OXPHOS OR "electron transport chain" OR "respiratory chain" OR "ATP production" OR "mitochondrial membrane potential" OR mtDNA OR "mitochondrial DNA" OR "mitochondrial biogenesis" OR "PGC-1 alpha" OR "PGC-1α" OR TFAM OR "citrate synthase" OR "complex I" OR "complex II" OR "complex III" OR "complex IV" OR "complex V" OR "mitochondrial ROS") AND (random* OR placebo OR trial OR intervention* OR treatment* OR therap* OR exercise OR training OR diet* OR nutrition* OR lifestyle OR "physical activity" OR pharmacological OR drug* OR medication* OR supplement*))

Scopus

TITLE-ABS-KEY (("type 2 diabetes" OR "type 2 diabetic" OR T2DM OR T2D OR NIDDM OR "non-insulin-dependent diabetes") AND (mitochondria* OR mitochondrial OR "mitochondrial dysfunction" OR "mitochondrial function" OR "mitochondrial respiration" OR "oxidative phosphorylation" OR OXPHOS OR "electron transport chain" OR "ATP production" OR "mitochondrial membrane potential" OR mtDNA OR "mitochondrial biogenesis" OR "PGC-1 alpha" OR TFAM OR "citrate synthase" OR "mitochondrial ROS") AND (random* OR placebo OR trial OR intervention* OR treatment* OR therap* OR exercise OR training OR diet* OR nutrition* OR lifestyle OR "physical activity" OR pharmacological OR drug* OR medication* OR supplement*))

supplementary search 2: Supplementary intervention-specific searches

In addition to the primary search strategy combining type 2 diabetes terms, mitochondrial-related terms, and randomized/interventional study design terms, supplementary intervention-specific searches will be conducted to improve the sensitivity of the search for prespecified pharmacological, nutritional, and lifestyle interventions with plausible mitochondrial relevance. Each supplementary search will combine four concepts: type 2 diabetes mellitus, mitochondrial-related terms, randomized or interventional study design terms, and intervention-specific terms. All records retrieved through supplementary searches will be deduplicated against the primary search results and screened using the same eligibility criteria. These searches are designed to improve sensitivity and will not expand the scope of eligible populations, study designs, interventions, or outcomes.

Prespecified intervention terms will include：

Pharmaceuticals： metformin； SGLT2 inhibitor； sodium-glucose cotransporter 2 inhibitor； empagliflozin；dapagliflozin；canagliflozin；ertugliflozin；GLP-1 receptor agonist；liraglutide；semaglutide；exenatide；dulaglutide；thiazolidinedione；pioglitazone；rosiglitazone

Nutrition/Supplements：coenzyme Q10；ubiquinone；ubiquinol；MitoQ；alpha-lipoic acid；α-lipoic acid；lipoic acid；nicotinamide riboside；nicotinamide mononucleotide；NAD precursor；NAD+ precursor； nicotinamide adenine dinucleotide

Lifestyle Categories：aerobic exercise；resistance training；strength training；high-intensity interval training； HIIT； caloric restriction； calorie restriction； energy restriction； ketogenic diet； time-restricted eating； time-restricted feeding； intermittent fasting

PubMed：

#1 Type 2 diabetes
("Diabetes Mellitus, Type 2"[Mesh] OR "type 2 diabetes"[tiab] OR "type II diabetes"[tiab] OR T2DM[tiab] OR "non insulin dependent diabetes"[tiab] OR "non-insulin-dependent diabetes"[tiab] OR "adult onset diabetes"[tiab])
#2 Mitochondrial-related terms
("Mitochondria"[Mesh] OR mitochondria*[tiab] OR mitochondrial[tiab] OR "mitochondrial dysfunction"[tiab] OR "mitochondrial function"[tiab] OR "mitochondrial respiration"[tiab] OR "oxidative phosphorylation"[tiab] OR OXPHOS[tiab] OR "electron transport chain"[tiab] OR "respiratory chain"[tiab] OR "mitochondrial biogenesis"[tiab] OR mtDNA[tiab] OR "mitochondrial DNA"[tiab] OR "citrate synthase"[tiab] OR "ATP production"[tiab] OR "reactive oxygen species"[tiab] OR "oxidative stress"[tiab] OR mitophagy[tiab])
#3 RCT/intervention terms
(randomized controlled trial[pt] OR controlled clinical trial[pt] OR randomized[tiab] OR randomised[tiab] OR randomly[tiab] OR placebo[tiab] OR trial[tiab])
#4 Prespecified intervention terms
(metformin[tiab] OR "SGLT2 inhibitor"[tiab] OR "SGLT2 inhibitors"[tiab] OR "sodium-glucose cotransporter 2 inhibitor"[tiab] OR empagliflozin[tiab] OR dapagliflozin[tiab] OR canagliflozin[tiab] OR ertugliflozin[tiab] OR "GLP-1 receptor agonist"[tiab] OR "glucagon-like peptide-1 receptor agonist"[tiab] OR liraglutide[tiab] OR semaglutide[tiab] OR exenatide[tiab] OR dulaglutide[tiab] OR thiazolidinedione*[tiab] OR pioglitazone[tiab] OR rosiglitazone[tiab] OR "coenzyme Q10"[tiab] OR ubiquinone[tiab] OR ubiquinol[tiab] OR MitoQ[tiab] OR

"alpha-lipoic acid"[tiab] OR "α-lipoic acid"[tiab] OR "lipoic acid"[tiab] OR "nicotinamide riboside"[tiab] OR "nicotinamide mononucleotide"[tiab] OR "NAD precursor"[tiab] OR "NAD+ precursor"[tiab] OR "aerobic exercise"[tiab] OR "resistance training"[tiab] OR "strength training"[tiab] OR "high-intensity interval training"[tiab] OR HIIT[tiab] OR "caloric restriction"[tiab] OR "calorie restriction"[tiab] OR "ketogenic diet"[tiab] OR "time-restricted eating"[tiab] OR "time-restricted feeding"[tiab] OR "intermittent fasting"[tiab])
#5 Final supplementary search
#1 AND #2 AND #3 AND #4 NOT (animals[mh] NOT humans[mh])

EMBASE via Ovid

1. 'type 2 diabetes mellitus'/exp OR 'type 2 diabetes':ti,ab,kw OR 'type II diabetes':ti,ab,kw OR T2DM:ti,ab,kw OR 'non insulin dependent diabetes':ti,ab,kw OR 'non-insulin-dependent diabetes':ti,ab,kw
2. 'mitochondrion'/exp OR mitochondria*:ti,ab,kw OR mitochondrial:ti,ab,kw OR 'mitochondrial dysfunction':ti,ab,kw OR 'mitochondrial function':ti,ab,kw OR 'mitochondrial respiration':ti,ab,kw OR 'oxidative phosphorylation':ti,ab,kw OR OXPHOS:ti,ab,kw OR 'electron transport chain':ti,ab,kw OR 'respiratory chain':ti,ab,kw OR 'mitochondrial biogenesis':ti,ab,kw OR mtDNA:ti,ab,kw OR 'mitochondrial DNA':ti,ab,kw OR 'mitochondrial membrane potential':ti,ab,kw OR 'citrate synthase':ti,ab,kw OR 'ATP production':ti,ab,kw OR 'reactive oxygen species':ti,ab,kw OR 'oxidative stress':ti,ab,kw OR mitophagy:ti,ab,kw
3. 'randomized controlled trial'/exp OR 'controlled clinical trial'/exp OR random*:ti,ab,kw OR placebo*:ti,ab,kw OR trial:ti,ab,kw OR intervention*:ti,ab,kw
4. 'metformin'/exp OR metformin:ti,ab,kw OR 'sodium glucose cotransporter 2 inhibitor'/exp OR 'SGLT2 inhibitor*':ti,ab,kw OR empagliflozin:ti,ab,kw OR dapagliflozin:ti,ab,kw OR canagliflozin:ti,ab,kw OR ertugliflozin:ti,ab,kw OR 'glucagon like peptide 1 receptor agonist'/exp OR 'GLP-1 receptor agonist*':ti,ab,kw OR liraglutide:ti,ab,kw OR semaglutide:ti,ab,kw OR exenatide:ti,ab,kw OR dulaglutide:ti,ab,kw OR 'thiazolidinedione'/exp OR thiazolidinedione*:ti,ab,kw OR pioglitazone:ti,ab,kw OR rosiglitazone:ti,ab,kw OR 'coenzyme q10'/exp OR 'coenzyme Q10':ti,ab,kw OR ubiquinone:ti,ab,kw OR ubiquinol:ti,ab,kw OR MitoQ:ti,ab,kw OR 'alpha lipoic acid'/exp OR 'alpha-lipoic acid':ti,ab,kw OR 'lipoic acid':ti,ab,kw OR 'nicotinamide riboside':ti,ab,kw OR 'nicotinamide mononucleotide':ti,ab,kw OR 'NAD precursor*':ti,ab,kw OR 'aerobic exercise':ti,ab,kw OR 'resistance training':ti,ab,kw OR 'strength training':ti,ab,kw OR 'high intensity interval training':ti,ab,kw OR HIIT:ti,ab,kw OR 'caloric restriction':ti,ab,kw OR 'calorie restriction':ti,ab,kw OR 'ketogenic diet':ti,ab,kw OR 'time restricted eating':ti,ab,kw OR 'time restricted feeding':ti,ab,kw OR 'intermittent fasting':ti,ab,kw
5. 1 AND 2 AND 3
6. 1 AND 2 AND 3 AND 4
7. 5 OR 6
8. limit 7 to human

CENTRAL

#1 MeSH descriptor: [Diabetes Mellitus, Type 2] explode all trees
#2 ("type 2 diabetes" OR "type II diabetes" OR T2DM OR "non-insulin-dependent diabetes" OR "adult onset diabetes"):ti,ab,kw

#3 #1 OR #2
#4 MeSH descriptor: [Mitochondria] explode all trees
#5 (mitochondria* OR mitochondrial OR "mitochondrial dysfunction" OR "mitochondrial function" OR "mitochondrial respiration" OR "oxidative phosphorylation" OR OXPHOS OR "electron transport chain" OR "respiratory chain" OR "mitochondrial biogenesis" OR mtDNA OR "mitochondrial DNA" OR "citrate synthase" OR "ATP production" OR "reactive oxygen species" OR "oxidative stress" OR mitophagy):ti,ab,kw
#6 #4 OR #5
#7 #3 AND #6
#8 (metformin OR "SGLT2 inhibitor" OR "SGLT2 inhibitors" OR empagliflozin OR dapagliflozin OR canagliflozin OR ertugliflozin OR "GLP-1 receptor agonist" OR liraglutide OR semaglutide OR exenatide OR dulaglutide OR thiazolidinedione OR pioglitazone OR rosiglitazone OR "coenzyme Q10" OR ubiquinone OR ubiquinol OR MitoQ OR "alpha-lipoic acid" OR "lipoic acid" OR "nicotinamide riboside" OR "nicotinamide mononucleotide" OR "NAD precursor" OR "NAD+ precursor" OR "aerobic exercise" OR "resistance training" OR "strength training" OR "high-intensity interval training" OR HIIT OR "caloric restriction" OR "calorie restriction" OR "ketogenic diet" OR "time-restricted eating" OR "time-restricted feeding" OR "intermittent fasting"):ti,ab,kw

#9 #3 AND #6 AND #8

Web of Science Core Collection

TS=(("type 2 diabetes" OR "type II diabetes" OR T2DM OR "non-insulin-dependent diabetes" OR "adult onset diabetes") AND (mitochondria* OR mitochondrial OR "mitochondrial dysfunction" OR "mitochondrial function" OR "mitochondrial respiration" OR "oxidative phosphorylation" OR OXPHOS OR "electron transport chain" OR "respiratory chain" OR "mitochondrial biogenesis" OR mtDNA OR "mitochondrial DNA" OR "citrate synthase" OR "ATP production" OR "reactive oxygen species" OR "oxidative stress" OR mitophagy) AND (random* OR placebo* OR trial OR "clinical trial" OR intervention*) AND (metformin OR "SGLT2 inhibitor*" OR empagliflozin OR dapagliflozin OR canagliflozin OR ertugliflozin OR "GLP-1 receptor agonist*" OR liraglutide OR semaglutide OR exenatide OR dulaglutide OR thiazolidinedione* OR pioglitazone OR rosiglitazone OR "coenzyme Q10" OR ubiquinone OR ubiquinol OR MitoQ OR "alpha-lipoic acid" OR "lipoic acid" OR "nicotinamide riboside" OR "nicotinamide mononucleotide" OR "NAD precursor*" OR "aerobic exercise" OR "resistance training" OR "strength training" OR "high-intensity interval training" OR HIIT OR "caloric restriction" OR "calorie restriction" OR "ketogenic diet" OR "time-restricted eating" OR "time-restricted feeding" OR "intermittent fasting"))

Scopus

TITLE-ABS-KEY (("type 2 diabetes" OR "type II diabetes" OR T2DM OR "non-insulin-dependent diabetes" OR "adult onset diabetes") AND (mitochondria* OR mitochondrial OR "mitochondrial dysfunction" OR "mitochondrial function" OR "mitochondrial respiration" OR "oxidative phosphorylation" OR OXPHOS OR "electron transport chain" OR "respiratory chain" OR "mitochondrial biogenesis" OR mtDNA OR "mitochondrial DNA" OR

"citrate synthase" OR "ATP production" OR "reactive oxygen species" OR "oxidative stress" OR mitophagy) AND (random* OR placebo* OR trial OR "clinical trial" OR intervention*) AND (metformin OR "SGLT2 inhibitor*" OR empagliflozin OR dapagliflozin OR canagliflozin OR ertugliflozin OR "GLP-1 receptor agonist*" OR liraglutide OR semaglutide OR exenatide OR dulaglutide OR thiazolidinedione* OR pioglitazone OR rosiglitazone OR "coenzyme Q10" OR ubiquinone OR ubiquinol OR MitoQ OR "alpha-lipoic acid" OR "lipoic acid" OR "nicotinamide riboside" OR "nicotinamide mononucleotide" OR "NAD precursor*" OR "aerobic exercise" OR "resistance training" OR "strength training" OR "high-intensity interval training" OR HIIT OR "caloric restriction" OR "calorie restriction" OR "ketogenic diet" OR "time-restricted eating" OR "time-restricted feeding" OR "intermittent fasting"))
